# Discovery of Monoacylglycerol Lipase (MAGL) Inhibitors Based on a Pharmacophore-Guided Virtual Screening Study

**DOI:** 10.3390/molecules26010078

**Published:** 2020-12-26

**Authors:** Vibhu Jha, Marzia Biagi, Valeria Spinelli, Miriana Di Stefano, Marco Macchia, Filippo Minutolo, Carlotta Granchi, Giulio Poli, Tiziano Tuccinardi

**Affiliations:** Department of Pharmacy, University of Pisa, Via Bonanno 6, 56126 Pisa, Italy; vibhu.jha@farm.unipi.it (V.J.); marziabiagi@outlook.com (M.B.); madavale83@libero.it (V.S.); miriana.distefano@phd.unipi.it (M.D.S.); marco.macchia@unipi.it (M.M.); filippo.minutolo@unipi.it (F.M.); carlotta.granchi@unipi.it (C.G.)

**Keywords:** virtual screening, MAGL, pharmacophore model, hit identification

## Abstract

Monoacylglycerol lipase (MAGL) is an important enzyme of the endocannabinoid system that catalyzes the degradation of the major endocannabinoid 2-arachidonoylglycerol (2-AG). MAGL is associated with pathological conditions such as pain, inflammation and neurodegenerative diseases like Parkinson’s and Alzheimer’s disease. Furthermore, elevated levels of MAGL have been found in aggressive breast, ovarian and melanoma cancer cells. Due to its different potential therapeutic implications, MAGL is considered as a promising target for drug design and the discovery of novel small-molecule MAGL inhibitors is of great interest in the medicinal chemistry field. In this context, we developed a pharmacophore-based virtual screening protocol combined with molecular docking and molecular dynamics simulations, which showed a final hit rate of 50% validating the reliability of the in silico workflow and led to the identification of two promising and structurally different reversible MAGL inhibitors, VS1 and VS2. These ligands represent a valuable starting point for structure-based hit-optimization studies aimed at identifying new potent MAGL inhibitors.

## 1. Introduction

The endocannabinoid system (ECS) is composed of the two cannabinoid receptors CB1 and CB2, the endogenous cannabinoid ligands (endocannabinoids, eCBs) *N*-arachidonoylethanolamine (anandamide, AEA) and 2-arachidonoylglycerol (2-AG), and the enzymes involved in endocannabinoid synthesis and degradation [1]. 2-AG is broadly considered as a major eCB, as it is about 100–1000 fold more abundant than AEA in the brain and peripheral organs [2]. 2-AG fully activates CB1 and CB2 receptors as an agonist, while AEA acts as partial agonist for both receptors [3]. The cannabinoid ligands AEA and 2-AG are synthesized on-demand from phospholipid precursors of the plasma membrane and released into the extracellular environment via facilitated diffusion. Fatty acid amide hydrolase (FAAH) is the enzyme that principally accounts for intracellular degradation of AEA, whereas monoacylglycerol lipase (MAGL) and α/β hydrolase-6 and -12 (ABHD6 and ABHD12) are involved in the intracellular degradation of 2-AG [4]. MAGL is responsible for the hydrolysis of about 85% of 2-AG in the brain and it is thus considered as the main hydrolytic enzyme of 2-AG [5,6]. MAGL-catalyzed hydrolysis of 2-AG leads to the formation of arachidonic acid (AA), which constitutes the precursor of eicosanoids that promote neuroinflammation and are involved in neurodegenerative diseases such as Parkinson’s and Alzheimer’s disease [7]. The modulation of the 2-AG levels obtained via MAGL inhibition is considered as a promising pharmacological strategy to activate the ECS without the typical side effects associated with direct CB1 receptor agonists. Furthermore, MAGL plays a key role in the development and spread of cancer, as it was found to be upregulated in aggressive cancer cells and primary tumors. MAGL-mediated hydrolysis of monoacylglycerols in adipose tissue and liver leads to the production of free fatty acids that are used by rapidly growing tumor cells as building blocks for the formation of new cellular membranes and for the synthesis of pro-tumorigenic signaling factors [8]. Due to the broad-spectrum of its therapeutic implications, MAGL has drawn increased attention as a promising drug target [9] and the therapeutic potential of selective MAGL inhibitors has been highlighted in animal models of inflammation, pain, anxiety and other neuroinflammatory diseases [10,11]. A significant number of MAGL inhibitors have been reported till date; however, most of them are characterized as irreversible inhibitors that form a covalent bond with MAGL. Several studies suggested that the genetic deletion or chronic inhibition of MAGL by irreversible MAGL inhibitors results in desensitization of CB1 receptors and ultimately impairs the benefits associated to the indirect CB1 stimulation [12,13]. Therefore, the identification of reversible MAGL inhibitors represents the basis for the development of an alternative promising therapeutic approach able to circumvent the drawbacks associated with irreversible MAGL inhibitors. In the last decades, many academic research groups and pharmaceutical industries have focused their research on the discovery of new MAGL inhibitors [14,15,16,17,18].

In this context, we developed a pharmacophore-based virtual screening (VS) protocol aimed at the identification of new reversible MAGL inhibitors, which was based on the latest X-ray structure of MAGL in complex with a reversible non-covalent inhibitor. Our vs. study, which combined pharmacophore screening, molecular docking with multiple approaches and MD simulations led to the discovery of two novel compounds endowed with micromolar inhibitory activity against MAGL, thus confirming the reliability of the vs. workflow and providing a starting point for future structure-based hit optimization studies aimed at designing new and more potent MAGL inhibitors.

## 2. Results

**Figure 1:**
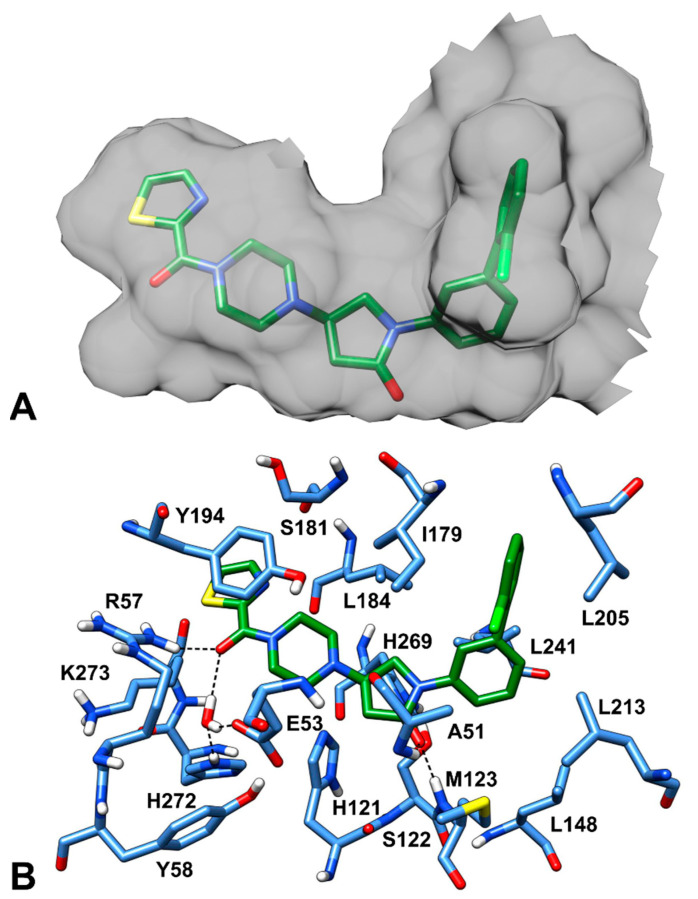
Piperazinyl-pyrrolidine inhibitor **3l** in the MAGL binding site (PDB code: 5ZUN). (**A**) The inner surface of the protein surrounding the ligand (green sticks), displaying the shape of the ligand-binding cavity, is shown in gray. (**B**) The protein residues surrounding the ligand, constituting the binding site, are show in blue sticks, while hydrogen bonds are shown as black dashed lines.

The latest X-ray structure of human MAGL in complex with the piperazinyl-pyrrolidine **3l** reversible inhibitor (Figure 1, PDB code: 5ZUN) [16] was used as a reference in the present study. The X-ray complex shows that the co-crystallized inhibitor forms key H-bond and hydrophobic interactions within MAGL binding site (Figure 1). The piperazine-carbonyl-thiazole portion of the inhibitor nicely occupies the bottom of the receptor pocket, while the biphenyl-pyrrolidin-2-one moiety resides at the entrance of the hydrophobic tunnel. The pyrrolidine carbonyl of the co-crystallized inhibitor establishes H-bond interactions with the backbone nitrogen of A51 and M123, within the oxyanion hole of MAGL catalytic site, that are considered as fundamental for MAGL inhibitory activity. Furthermore, the carbonyl group linked to the thiazole ring of the ligand forms an H-bond with R57; notably, the same carbonyl group also shows an interaction with a structural water molecule that forms an H-bond network with E53 and H272. These water-bridged H-bonds between the co-crystallized inhibitor and the two residues are supposed to contribute to the strong binding affinity for the target. A significant number of hydrophobic interactions were also noted between the co-crystallized ligand and the enzyme binding site. The phenyl ring of the inhibitor is encircled by the sidechains of A51, I179, L213 and L241, establishing strong van der Waals contacts with these residues. The terminal *o*-chlorophenyl ring of the inhibitor forms hydrophobic contacts with I179 and L205, while the electron-rich chlorine was found to be in proximity to I179, L205 and L213, thus forming additional van der Waals interactions. Finally, the thiazole ring of the inhibitor forms a face-to-face π-π stacking with Y194 that contributes to the ligand inhibitory activity.

After analyzing the main protein-ligand interactions in the enzyme binding site, as a first step of our study, the X-ray structure of MAGL in complex with its piperazinyl-pyrrolidine inhibitor was subjected to a preliminary MD simulation of 20 ns in order to evaluate the stability of the above-mentioned H-bond interactions, with particular attention to the H-bond network mediated by the structural water molecule. In fact, the H-bond interaction between the thiazole carbonyl and the structural water was believed to be of remarkable importance, due to the presence of this water molecule in various MAGL X-ray structures [19]. Therefore, we envisioned that targeting the structural water mediating the H-bond network with E53 and H272 could provide a novel strategy for the development of potent and reversible MAGL inhibitors. The MD analysis highlighted a remarkable stability of the ligand disposition throughout the simulation, possessing an average RMSD of 0.4 Å with respect to the starting coordinates and thus well maintaining the hydrophobic and van der Waals interactions identified in the X-ray complex. Similarly, the structural water was found to maintain its disposition, with an average RMSD of 0.5 Å during the MD (Figure S1). The H-bond interactions of the ligand with A51 and M123 were maintained for more than 85% of the MD, whereas the interactions with R57 and the structural water molecule were observed for about 70% of the MD.

**Figure 2:**
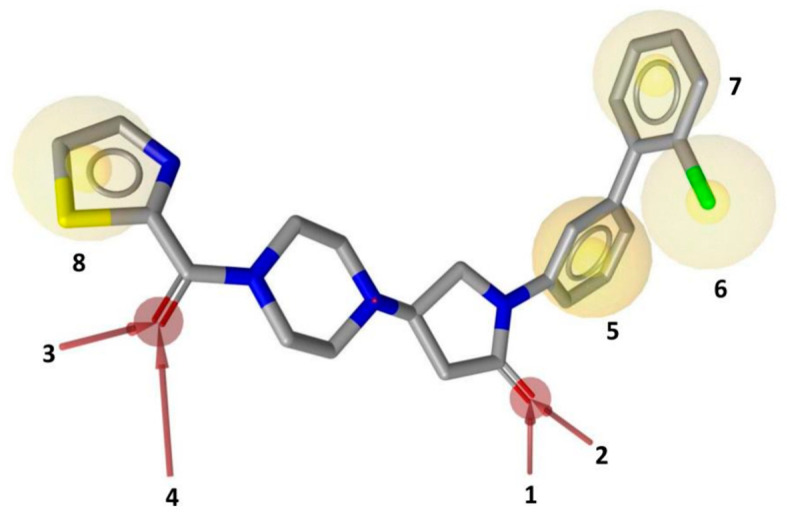
Receptor-based pharmacophore model with mandatory features 1–5 and optional features 6–8 superimposed with MAGL inhibitor **3l** in the X-ray complex.

The MD simulation thus confirmed the stability of all the interactions observed in the reference ligand-protein X-ray structure, which were subsequently exploited to develop a pharmacophore model. A receptor-based pharmacophore model was constructed using Ligandscout program [20], considering both ligand-protein interactions and the excluded volume of the binding pocket. A total of eight pharmacophore features were considered, including five mandatory and three optional features (Figure 2). The four H-bond acceptor features corresponding to the interactions of both ligand’s carbonyl groups with A51, M123, R57 and the structural water (Figure 1) were set as mandatory. A hydrophobic feature representing the phenyl ring of the ligand involved in van der Waals contacts with A51, I179, L213 and L241 residues, was considered as the fifth mandatory feature. The three optional features of the pharmacophore included three additional hydrophobic features representing: (a) the chlorine of the co-crystallized inhibitor forming van der Waals contacts with I179, L205 and L213, (b) the terminal phenyl ring showing hydrophobic contacts with I179 and L205, (c) the ligand thiazole ring exhibiting face-to-face π-π stacking with Y194 (Figure 2).

The pharmacophore model was screened against a combination of four commercial databases (i.e., Enamine, ChemBridge, Pharmeks and Vitas-M) that comprise around 4 million compounds. The pharmacophore screening step allowed to select a large set of diverse compounds showing the desirable protein-ligand interactions in the MAGL binding site, which were ranked according to the number of satisfied features. In total, 276,150 compounds matched at least the 5 mandatory features of the pharmacophore, while 5707 molecules showed to satisfy all 8 features of the model (Table 1). Given the large number of pharmacophore hits identified, only those matching at least seven features were selected for the subsequent stage of the vs. workflow.

The next step was the validation of the docking procedures, which involved the assessment of various programs to identify the methods able to reproduce the binding orientation of the X-ray ligand. The co-crystallized inhibitor **3l** was thus subjected to self-docking studies using 13 docking methods. The root mean-square deviation (RMSD) between the position of the co-crystallized ligand predicted by docking and its experimental disposition was calculated. The comprehensive results of preliminary self-docking studies on 5ZUN X-ray structure are shown in Table 2: 9 out of 13 docking methods showed good results, with RMSD values below 2.0 Å.

The docking solutions were also visually checked to confirm that the fundamental ligand-protein interactions with A51, R57, M123 and the structural water molecule within MAGL binding pocket were maintained. After this analysis, only 6 docking methods (i.e., Dock6, Glamdock, Glide SP and Gold with ASP, PLP and Goldscore fitness functions) were selected for the next steps, since only the binding poses produced with these procedures showed all fundamental ligand-protein interactions. The 19,314 compounds retrieved from the pharmacophore screening were eventually docked in the X-ray structure of MAGL using the six selected docking methods. The top ranked docking solutions produced for each ligand by each docking method were subjected to a pose filtering analysis in search for compounds maintaining the interactions represented by the pharmacophore model. In particular, only compounds forming the H-bonds with A51, R57, M123 and the structural water molecule and respecting at least two out of the four hydrophobic features, where retained. By intersecting the six different subsets of compounds obtained by filtering the results of the six different docking methods, we identified 113 compounds showing the desired interactions in the binding poses predicted by all methods. Finally, these ligands were subjected to a pose consensus analysis in which the six docking results obtained for each compound were clustered together in search for common binding modes [21,22]. Only the 40 compounds for which a full pose consensus among all six docking procedures was observed (i.e., compounds for which all predicted binding modes had reciprocal RMSDs below 2.0 Å) were selected and considered as potential MAGL inhibitors. The selected compounds were then subjected to 20 ns of MD simulations with the same parameters and conditions as used in the preliminary MD study of the reference X-ray complex. The Gold Goldscore pose of each ligand was used as the starting ligand disposition within MAGL binding site, since Goldscore procedure showed the best RMSD value in the preliminary self-docking analysis. The trajectories of the 40 protein-ligand complexes were analyzed in terms of H-bond stability and ligand RMSD with respect to the starting coordinates. The compounds that showed an average RMSD value lower than 2.0 Å and the maintenance of the H-bonds with R57, the structural water molecule and at least A51 or M123 for more than 70% of the simulation were retained. Out of the 40 analyzed compounds, seven ligands were ultimately selected by the MD filter and thus subjected to enzyme inhibition assays in order to test their inhibitory activity against MAGL (see Materials and methods for details). As shown in Table 3, four compounds out of the seven tested showed a MAGL inhibition activity; considering the high similarity between **VS2** and **VS4**, the vs. study results in a hit rate of 50% with the two best MAGL inhibitors (i.e., **VS1** and **VS2**) with an IC_50_ of 34.7 µM and 48.9 µM. A similarity search for compounds **VS1** and **VS2** against the already published hydrolase inhibitors revealed that there are no hydrolase inhibitors with a similarity score greater than 80 (score = 100 means that the two compounds are identical).

**Figure 3:**
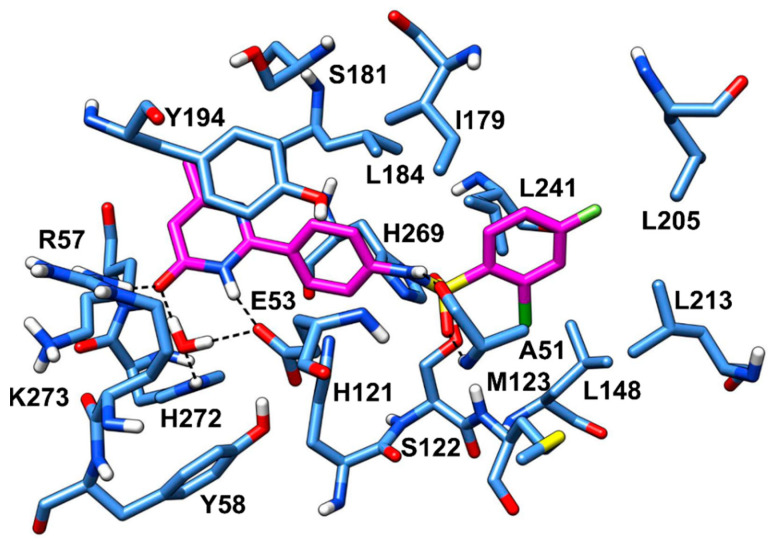
Minimized average structure of **VS1** within MAGL binding site.

Figure 3 shows the minimized average structure of compound **VS1** in complex with MAGL obtained by the last 10 ns of MD simulation. The sulphonamide moiety of **VS1** displays multiple H-bond interactions in the oxyanion hole of MAGL binding site. The sulfonamide oxygen of the ligand forms an H-bond with the backbone nitrogen of A51 observed for 82% of the simulation. Although the interaction with the backbone nitrogen of M123 was lost during the MD, the sulphonamide nitrogen of the ligand establishes an additional stable H-bond with the backbone oxygen of A51 maintained for 97% of the MD, which effectively anchors the ligand core to the protein binding site. The carbonyl oxygen of the ligand pyrimidinone moiety forms an H-bond interaction with R57 maintained for 98% of the simulation and an H-bond network with the structural water molecule, E53 and H272 observed for 94% of the MD. Moreover, the pyrimidinone nitrogen forms an additional direct H-bond interaction with E53 maintained throughout the whole simulation. Finally, the *o*-chloro-*p-*fluorophenyl ring of **VS1** shows van der Waals contacts with L205, L213, L241 and A51, while a face-to-face π-π stacking is observed between the pyrimidinone ring of **VS1** and Y194. These hydrophobic interactions, which well mimic those identified in the reference X-ray structure, most likely contribute to the binding affinity of the ligand for MAGL.

**Figure 4:**
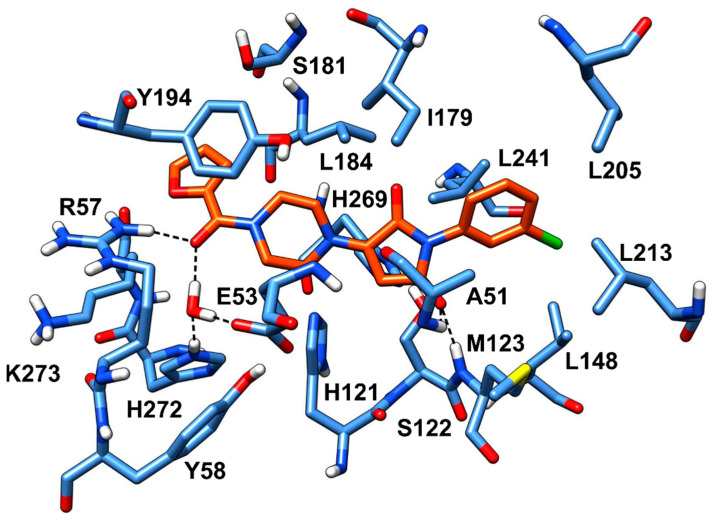
Minimized average structure of **VS2** within MAGL binding site.

Besides **VS1**, the hit compound **VS2** showed a promising MAGL inhibitory activity with an IC_50_ below 50 µM and also **VS3** and **VS4** demonstrated MAGL inhibition with IC_50_ values of 79.4 µM and 138 µM, respectively (Table 3). As shown in Figure 4, the carbonyl group of **VS2** core ring establishes both H-bonds with A51 and M123, maintained for 95% and 74%, respectively, during the simulation. The furan carbonyl of **VS2** is involved in H-bond interactions with R57 and the structural water molecule observed for more than 80% of the simulation, whereas the water-bridged interactions with E53 and H272 were maintained for about 70% of the MD. The *m*-chlorophenyl ring of the inhibitor forms van der Waals contacts with L213, L241 residues while the furan ring shows a π-π stacking with Y194 (Figure 4). The possible explanation behind the lower IC_50_ value of **VS1** as compared to **VS2**, could be ascribed to the presence of a direct H-bond between **VS1** and the carboxyl group of E53, which is instead absent in the binding mode predicted for **VS2**. Compounds **VS3** and **VS4** show a predicted pattern of ligand-protein interactions like that described for **VS2**. However, both **VS3** and **VS4** show only a single H-bond with the residues of the oxyanion hole (Figure S2), which can probably explain their lower inhibitory activity with respect to **VS2**.

**Figure 5:**
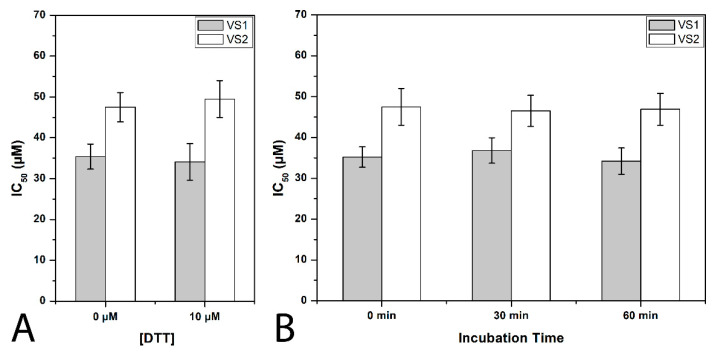
Compound **VS1**- and **VS2**-MAGL inhibition analysis. (**A**) Effect of DTT on MAGL inhibition activity. (**B**) IC_50_ (µM) values of **VS1** and **VS2** at different preincubation times with MAGL (0 min, 30 min and 60 min).

In order to verify whether the most promising compounds could interact with cysteine residues of MAGL enzyme, the activity of the two most potent inhibitors **VS1** and **VS2** was also tested in presence of the thiol-containing agent 1,4-dithio-D,L-threitol (DTT). As shown in Figure 5, the IC_50_ value of the two compounds was only very slightly, but not significantly, influenced by the presence of DTT, shifting for **VS1** from 35.4 µM when assayed in the absence of DTT to 34.1 µM when assayed in the presence of 10 µM DTT and for **VS2** from 47.5 µM to 49.5 µM, thus excluding any significant interaction of the two ligands with MAGL cysteine residues. Furthermore, with the aim of establishing whether the mechanism of inhibition was reversible or irreversible, the effects of preincubation on the inhibitory activity of compounds **VS1** and **VS2** were evaluated. In this assay, the compounds were preincubated with the enzyme for 0, 30 and 60 min before adding the substrate to start the enzymatic reaction. An irreversible inhibitor should show a higher potency after longer incubation times, whereas a reversible inhibitor should display a constant inhibition potency that is independent from the incubation time. As shown in Figure 5, this assay confirmed the reversible character of both compounds, as they did not show any significant increase in inhibitory potency at longer incubation times. Taken together, these experimental studies, aimed at better characterizing the mode of action of compounds **VS1** and **VS2**, further supported the reliability of the binding mode predicted for the two ligands.

## 3. Conclusions

A pharmacophore-based vs. protocol was developed and performed to identify new MAGL inhibitors. The key protein-ligand interactions detected from the latest X-ray structure of MAGL in complex with a non-covalent piperazinyl-pyrrolidine inhibitor were exploited to build a receptor-based pharmacophore model. In particular, an H-bond interaction with a structural water molecule was taken into consideration, as it was believed to contribute to the binding affinity of the reference MAGL inhibitor. In fact, many MAGL X-ray structures present a water molecule located in the polar portion of MAGL binding site that forms a network of interactions with E53 and H272. These two residues demonstrated to represent effective anchoring points for MAGL ligands, since compounds predicted to displace the structural water molecule and form direct interactions with E53 and H272, such as our reference compound **17b**, showed nanomolar MAGL inhibitory activity. However, compound **3l**, the co-crystallized ligand in the MAGL-inhibitor X-ray complex used as a reference for our vs. protocol, displayed interactions with E53 and H272 through an H-bond network mediated by the structural water molecule and showed a very high MAGL inhibitory potency. For this reason, we envisioned that such water molecule could be considered in our vs. in order to search for novel potential ligands that could exploit the structural water as an indirect anchoring point to MAGL binding site. The whole vs. workflow, comprising pharmacophore screening, multiple parallel molecular docking studies and a final MD simulation analysis led to the selection of seven final compounds that were tested for MAGL inhibition activity. Out of the seven selected molecules, two compounds showed a promising inhibitory potency (with IC_50_ below 50 µM) and two additional compounds demonstrated MAGL inhibition activity in the micromolar range. The positive outcomes from this in silico study provided validation of the reliability of the vs. workflow, which may be further applied to screen additional commercial databases for identifying new MAGL ligands. In particular, the obtained vs. result confirmed that targeting the structural water molecule within MAGL binding site, accounting for the H-bond network with E53 and H272, represents a profitable strategy for identifying novel hit compounds. The water-mediated H-bonds with E53 and H272 were confirmed to represent key MAGL-anchoring interactions that should be considered in structure-based drug design campaigns aimed at developing potent MAGL inhibitors. In this context, despite **VS1** and **VS2** are characterized by a micromolar MAGL inhibition, the discovery of these two structurally unrelated molecules could serve as a starting point for structure-based hit optimization studies aimed at designing new and more potent MAGL ligands.

## 4. Materials and Methods

### 4.1. Pharmacophore Model Generation

The pharmacophore model was constructed using LigandScout 4.2 [20]. The pharmacophore hypothesis was generated from the X-ray structure of human MAGL in complex with piperazinyl-pyrrolidine inhibitor (PDB code: 5ZUN) [16]. A complete model that represents all the possible pharmacophore features of the ligand, including the interaction with the structural water molecule, recognized by the program was generated; all the features were taken into consideration in the final pharmacophore model, for a total of four H-bond acceptor features and four hydrophobic features. Of these, five pharmacophore features (four H-bond and a hydrophobic feature) were set as mandatory and the other three (hydrophobic) features were set as optional. The excluded volume spheres that resemble regions of space in the proximity of the pharmacophore model occupied by the enzyme, were also taken into consideration for the pharmacophore model development.

### 4.2. Database Generation and Pharmacophore Screening

Approximately 4 million compounds from the commercial Enamine, Vitas-M, ChemBridge and Pharmeks databases were used as the screening database. The software iCon [23] implemented in LigandScout was used to carry out ligand conformational sampling and to set up the screening database. The generated pharmacophore model including five mandatory features, three optional features and the excluded volume spheres was used to screen the generated screening database and to search for desirable compounds. Only compounds matching at least five mandatory features of the model and respecting the excluded volume constraints were retrieved in this search.

### 4.3. Molecular Docking and Pose Filtering

All docking calculations were carried out using the X-ray structure of human MAGL in complex with piperazinyl-pyrrolidine inhibitor (PDB code: 5ZUN) [16] already employed for pharmacophore modeling. In total, 13 different docking procedures were used in this study: Autodock 4.2.3, Autodock Vina 1.1, Dock 6.7, Fred 3.0, GlamDock, Gold 5.1 with its four fitness functions (i.e., ChemScore, GoldScore, ChemPLP and Astex Statistical Potential), Glide 5.0 with standard precision (SP) and extra precision (XP) methods, Plants and rDock, following the procedures described in our previous studies [21,24]. A self-docking analysis for each docking method was performed by calculating the root-mean-square deviation (RMSD) between the position of the crystallographic ligand predicted by docking and its known experimental disposition, using the rms_analysis software of Gold suite. The filtering of the docking results was performed by superimposing the docked compounds to the pharmacophore model directly from the supplied poses, without changing their coordinates. The pose consensus analysis was performed using our in-house complete-linkage clustering procedure, as described in our precious studies [25]. 

### 4.4. Molecular Dynamics Simulations

All simulations were carried out using Amber 16 [26]. General amber force field (GAFF) parameters were assigned to the ligands, while partial charges were determined using the AM1-BCC method as implemented in the Antechamber suite of Amber 16. All ligand-protein complexes were placed in a rectangular parallelepiped water-box, by using TIP3P explicit solvent model and solvated with a 20.0 Å water cap. Either Na^+^ or Cl^−^ ions were added as counterions in order to neutralize the systems. Before MD simulations, two stages of energy minimization were carried out; in the first step, a position restraint of 100 kcal/(mol∙Å^2^) was applied to the complex, thus minimizing only the position of the water molecules through 5000 steps of steepest descent followed by conjugate gradient, until a convergence of 0.05 kcal/(mol·Å^2^). Successively, the whole system was energy minimized imposing a harmonic force constant of 10 kcal/(mol∙Å^2^) only on the protein α-carbons. The minimized complexes were used as starting conformations for the MD simulations. Periodic boundary conditions and particle mesh Ewald (PME) electrostatics were employed in the simulations. The time step of the simulations was 2 fs, a cut-off of 10.0 Å was set for the non-bonded interactions and SHAKE algorithm was used to keep all bonds involving hydrogen atoms rigid. Constant-volume MD simulation was performed for the first 0.5 ns, during which the temperature of the system was raised from 0 to 300 K. The system was then equilibrated through 3 ns of constant pressure periodic boundary MD, employing the Langevin thermostat in order to keep the temperature of the system constant. At last, additional 16.5 ns of constant pressure MD production were performed. Thus, a total of 20 ns MD simulation was carried out for each protein-ligand complex analyzed in this study. In all MD steps the α-carbons of the protein were restrained with a harmonic force constant of 10 kcal/mol·Å^2^. All the obtained MD trajectories were analyzed using the cpptraj program implemented in Amber 16 [27].

### 4.5. Similarity Search

The test compounds were compared with published hydrolase inhibitors. This comparison was made by using the Similarity Search task of SciFinder. Similarity search locates structures that are similar to the query, based on a two-dimensional small-molecule comparison using a Tanimoto similarity metric. A score of 100 means that the two structures are identical.

### 4.6. Enzymatic Assays

Compounds **VS1**–**VS7** were bought from their source databases (ChemBridge, Enamine, Pharmeks and Vitas-M corporation). Human recombinant MAGL and 4-nitrophenyl acetate substrate (4-NPA) were bought from Cayman Chemical (Ann Arbor, MI, USA). The reversible compound **17b** reported by Granchi et al. in 2016 was synthesized in our laboratory and used as reference compound [19]. Compound **3l** was synthesized in our laboratory according to the procedure reported by Aida et al. [16] and used as reference compound. The IC_50_ values were produced in 96-well microtiter plates. The MAGL reaction was carried out at rt at a final volume of 200 µL in 10 mM Tris buffer, pH 7.2, containing 1 mM EDTA and 0.1 mg/mL BSA. A total of 150 µL of 4-NPA 133.3 µM was added to 10 µL of DMSO containing the appropriate amount of compound. The reaction was started by adding 40 µL of MAGL (11 ng/well) in such a way that the assay was linear over 30 min. After the reaction had progressed for 30 min, absorbance values were measured by using a VictorX3 instrument (PerkinElmer, Waltham, MA, USA) at 405 nm. Two more reactions were carried out: one reaction containing no compounds and the second one containing neither enzyme nor inhibitor. To remove possible false positive results, a blank analysis was performed for each compound concentration and the final absorbance results were obtained detracting the absorbance produced by the presence of all the components except MAGL in the same conditions [28].

## Figures and Tables

**Table 1:** Results of the receptor-based pharmacophore screening.

Number of Matched Features	Number of Compounds
8	5707
7	19,314
6	182,236
5	276,150

**Table 2:** Preliminary self-docking analyses on 5ZUN X-ray structure.

Docking Method	RMSD (Å)
Autodock 4.2	1.5
Dock6	0.5
Fred	3.8
Glamdock	1.3
Glide SP	0.6
Glide XP	1.5
Gold ASP	1.4
Gold Chemscore	3.1
Gold Goldscore	0.6
Gold PLP	0.7
Plants	3.2
rDock	1.6
Vina	9.4

**Table 3:** Structure and MAGL inhibition activity of the tested compounds. Compound **17b** [19] and compound **3l** [16] were used as reference compounds.

#	Structure	IC_50_ (µM)
**VS1**	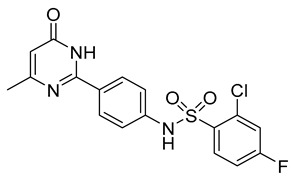	34.7 ± 2.4
**VS2**	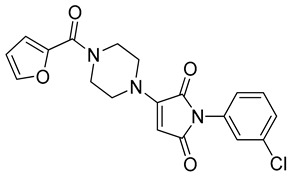	48.9 ± 4.2
**VS3**	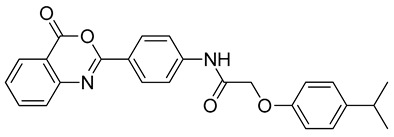	79.4 ± 6.3
**VS4**	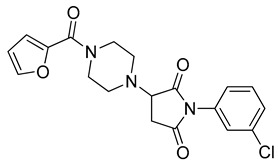	138 ± 11
**VS5**	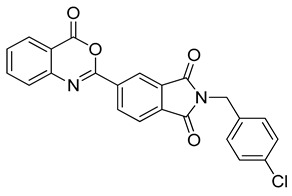	>200
**VS6**	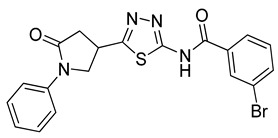	>200
**VS7**	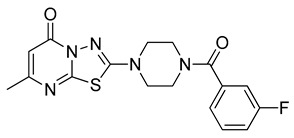	>200
**17b**	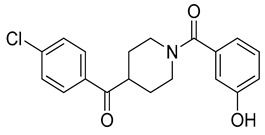	0.90 ± 0.08
**3l**	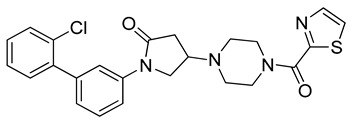	0.042 ± 0.001

## Data Availability

All data comes from the author.

## Supplementary Materials

The following are available online, Figure S1: MD simulation analysis of the reference X-ray complex, Figure S2: Predicted binding mode of VS3 and VS4 within MAGL.

## Sample Availability

Samples of the compounds are available from the authors.
