# Supplementary material for: Discovery of Monoacylglycerol Lipase (MAGL) Inhibitors Based on a Pharmacophore-Guided Virtual Screening Study
Source: Molecules. 2020 Dec 26;26(1):78. doi: 10.3390/molecules26010078 (PMC7794939; doi:10.3390/molecules26010078)
Supplement: Supplementary file 1 [file molecules-26-00078-s001.pdf]

Supporting Information

Discovery of monoacylglycerol lipase (MAGL) inhibitors based on a pharmacophore-guided virtual screening study

Vibhu Jha, Marzia Biagi, Valeria Spinelli, Miriana Di Stefano, Marco Macchia, Filippo Minutolo, Carlotta Granchi, Giulio Poli* and Tiziano Tuccinardi*

E-mail: tiziano.tuccinardi@unipi.it, giulio.poli@unipi.it

Table of Contents

Figure S1. MD simulation analysis of the reference X-ray complex **S2**

Figure S2. Predicted binding mode of VS3 and VS4 within MAGL **S2**

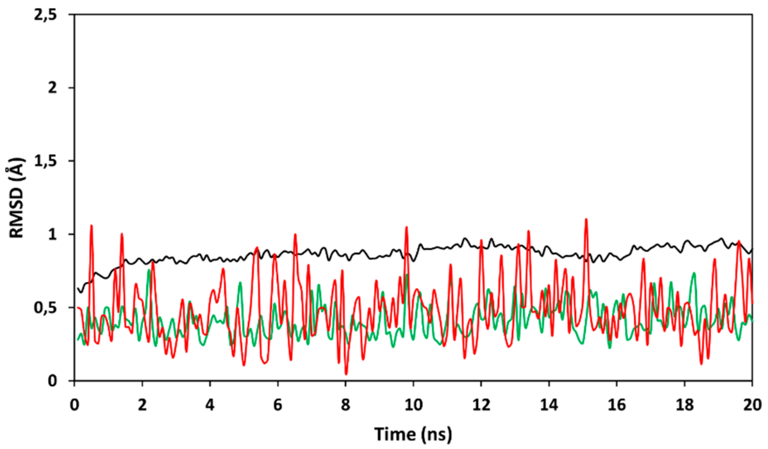

Figure S1. MD simulation analysis of the reference MAGL-inhibitor X-ray-complex (PDB code 5ZUN). The RMSD of the ligand during the 20 ns of simulation, with respect to the crystallographic coordinates, is shown in red, confirming the stability of the ligand binding mode (average RMSD = 0.4 Å). The RMSD of the structural water molecule during the MD is shown in green: the low RMSD fluctuation and the small average RMSD value (0.5 Å) indicate a strong stability of the water disposition and thus its importance for the ligand binding mode. Finally, the RMSD of the receptor heavy atoms during the MD is shown in black, indicating a high conformation stability (average RMSD = 0.9 Å) and thus confirming the reliability of the MD protocol.

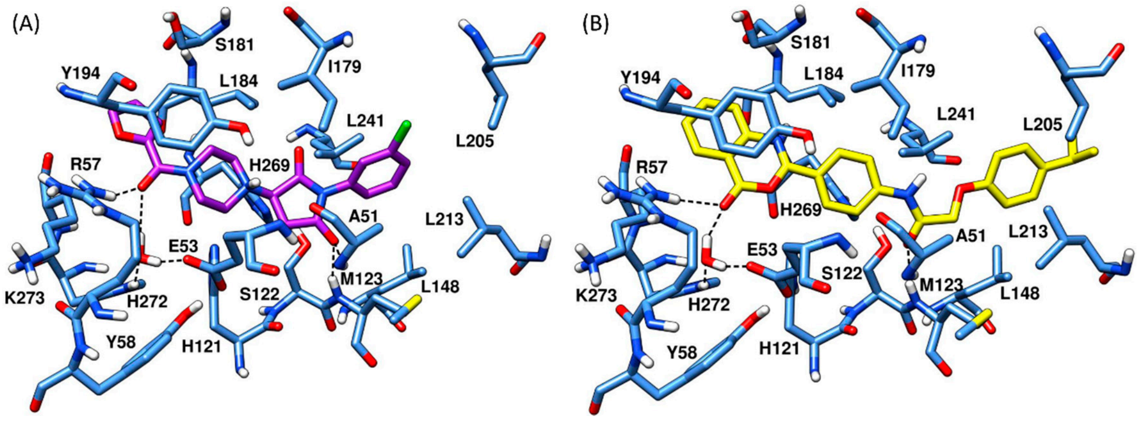

Figure S2. Minimized average structure of compounds **VS3** (A) and **VS4** (B) within MAGL binding site.
